# Re-expression of DIRAS3 and p53 induces apoptosis and impaired autophagy in head and neck squamous cell carcinoma

**DOI:** 10.1186/s40779-020-00275-3

**Published:** 2020-10-11

**Authors:** Zhe Liu, Douglas R. Hurst, Xing Qu, Li-Guang Lu, Chen-Zhou Wu, Yu-Yu Li, Yi Li

**Affiliations:** 1 State Key Laboratory of Oral Diseases & National Clinical Research Center for Oral Diseases, West China Hospital of Stomatology, Sichuan University, Chengdu, 610041 China; 2 Department of Head and Neck Oncology, West China Hospital of Stomatology, Sichuan University, Chengdu, 610041 China; 3 Department of Pathology, University of Alabama at Birmingham, Birmingham, AL 35216 USA; 4 Department of Evidence Based Stomatology, West China Hospital of Stomatology, Sichuan University, Chengdu, 610041 China

**Keywords:** DIRAS3, p53, Apoptosis, Autophagy, Head and neck squamous cell carcinoma

## Abstract

**Background:**

p53 and DIRAS3 are tumor suppressors that are frequently silenced in tumors. In this study, we sought to determine whether the concurrent re-expression of p53 and DIRAS3 could effectively induce head and neck squamous cell carcinoma (HNSCC) cell death.

**Methods:**

CAL-27 and SCC-25 cells were treated with Ad-DIRAS3 and rAd-p53 to induce re-expression of DIRAS3 and p53 respectively. The effects of DIRAS3 and p53 re-expression on the growth and apoptosis of HNSCC cells were examined by TUNEL assay, flow cytometric analysis and MTT. The effects of DIRAS3 and p53 re-expression on Akt phosphorylation, oncogene expression, and the interaction of 4E-BP1 with eIF4E were determined by real-time PCR, Western blotting and immunoprecipitation analysis. The ability of DIRAS3 and p53 re-expression to induce autophagy was evaluated by transmission electron microscopy, LC3 fluorescence microscopy and Western blotting. The effects of DIRAS3 and p53 re-expression on HNSCC growth were evaluated by using an orthotopic xenograft mouse model.

**Results:**

TUNEL assay and flow cytometric analysis showed that the concurrent re-expression of DIRAS3 and p53 significantly induced apoptosis (*P* < 0.001). MTT and flow cytometric analysis revealed that DIRAS3 and p53 re-expression significantly inhibited proliferation and induced cell cycle arrest (*P* < 0.001). Mechanistically, the concurrent re-expression of DIRAS3 and p53 down-regulated signal transducer and activation of transcription 3 (STAT3) and up-regulated p21^WAF1/CIP1^ and Bax (*P* < 0.001). DIRAS3 and p53 re-expression also inhibited Akt phosphorylation, increased the interaction of eIF4E with 4E-BP1, and reduced the expression of c-Myc, cyclin D1, vascular endothelial growth factor (VEGF), fibroblast growth factor (FGF), epidermal growth factor receptor (EGFR) and Bcl-2 (*P* < 0.001). Moreover, the concurrent re-expression of DIRAS3 and p53 increased the percentage of cells with GFP-LC3 puncta compared with that in cells treated with control adenovirus (50.00% ± 4.55% vs. 4.67% ± 1.25%, *P* < 0.001). LC3 fluorescence microscopy and Western blotting further showed that DIRAS3 and p53 re-expression significantly promoted autophagic activity but also inhibited autophagic flux, resulting in overall impaired autophagy. Finally, the concurrent re-expression of DIRAS3 and p53 significantly decreased the tumor volume compared with the control group in a HNSCC xenograft mouse model [(3.12 ± 0.75) mm^3^ vs. (189.02 ± 17.54) mm^3^, *P* < 0.001].

**Conclusions:**

The concurrent re-expression of DIRAS3 and p53 is a more effective approach to HNSCC treatment than current treatment strategies.

## Background

Head and neck squamous cell carcinoma (HNSCC) is the sixth most frequently diagnosed tumor, with an estimated annual worldwide incidence of 550,000 new cases [1]. Multiple factors, including hereditary predisposition, human papilloma virus (HPV) infection, alcohol consumption, tobacco use, and chronic mechanical trauma, are involved in the pathogenesis of HNSCC. These risk factors result in DNA damage, gene mutations, and consequently the malignant transformation of normal cells [2]. Gene therapy has been used to target genetic abnormalities, typically via the augmentation of silenced tumor suppressor genes or the blockage of activated oncogenes. Although many roadblocks have been encountered for the clinical success of gene therapy over the past several years, there remains excitement for the potential of many gene therapy approaches to improve personalized cancer therapy.

The mutation of the p53 tumor suppressor gene, which plays crucial functions in genomic stability, cell cycle regulation, stress-induced reaction and DNA repair, arises in 60–80% of patients with HNSCC [3, 4]. p53-based cancer therapies, including wild-type p53 gene transfer, p53 vaccines, MDM2 antagonist, and mutant p53 re-activation, are used clinically or are currently undergoing trials [5]. Gendicine, the world’s first gene therapy product approved by the Chinese State Food and Drug Administration (SFDA) for the treatment of advanced HNSCC in 2003, showed significant therapeutic benefits and improved survival rates in a number of trials [6]. Gendicine is a recombinant human serotype 5 adenovirus encoding human wild-type p53 gene (rAd-p53). It is typically used as an adjuvant therapy to chemotherapy, radiotherapy and surgery [7]. Improvement of the construction of recombinant adenovirus, or combined treatment with new agents that have synergistic antitumor effects, will undoubtedly enhance the therapeutic efficacy of p53-modulated gene therapy.

DIRAS family GTPase 3 (DIRAS3; also known as ARHI) encodes a 26 kD GTP-binding protein with 60% homology to Ras and Rap. Unlike the Ras and Rap oncogenes, DIRAS3 re-expression enhances apoptosis and autophagic cell death in ovarian and breast cancer cells, indicating that it could function as a tumor suppressor [8, 9]. The expression of DIRAS3 is frequently downregulated or abrogated in multiple cancers. Zhang et al. [10] evaluated DIRAS3 expression in paired adjacent normal and cancerous tongue tissue samples from 20 patients with tongue squamous cell carcinoma and found that DIRAS3 expression was significantly downregulated in cancerous tongue tissues. The re-expression of DIRAS3 led to retarded cell growth, angiogenesis, migration and invasion in breast, ovarian and liver cancer [11–13]. However, whether DIRAS3 re-expression can suppress tumor growth in HNSCC has not yet been studied.

Previously, we discussed the clinical application of p53 gene therapy using Gendicine for solid malignant tumors. We summarized the key points of drug administration, including the routes of administration, dosage calculation and treatment cycles, based on our clinical experience, as well as findings of some trials [14]. In the current study, we examined the effects of DIRAS3 and p53 re-expression by adenovirus infection in HNSCC cells. Concurrent re-expression of DIRAS3 and p53 induced apoptosis and cell cycle arrest and impaired autophagy. These phenotypes are likely the results of decreased Akt phosphorylation, increased interaction of 4E-BP1 with eIF4E, inhibition of oncogene expression, and autophagic vacuole accumulation. We tested the efficacy of combined re-expression of DIRAS3 and p53 in vivo and demonstrated significant suppression of human HNSCC xenograft tumor growth.

## Methods

### Antibodies and reagents

Gendicine was purchased from Shenzhen SiBiono (Shenzhen, China). Primary antibodies against DIRAS3, p53, Bax, signal transducer and activation of transcription 3 (STAT3), c-Myc, cyclin D1, vascular endothelial growth factor (VEGF), fibroblast growth factor (FGF), epidermal growth factor receptor (EGFR), Bcl-2, and GAPDH were purchased from Abcam (Cambridge, MA, USA). Primary antibodies against p21^WAF1/CIP1^, Akt, p-Ser473-Akt, p-Thr308-Akt, 4E-BP1, p-Thr37/46-4E-BP1, p-Ser65-4E-BP1, p-Thr70-4E-BP1, and LC3 were obtained from Cell Signaling Technology (Beverly, MA, USA). All secondary antibodies were purchased from Zhongshan Jinqiao Biotech (Beijing, China). Baf A1 was obtained from Sigma-Aldrich (St. Louis, MO, USA).

### Cell culture

CAL-27 and SCC-25 human head and neck squamous cell carcinoma cells were obtained from the American Type Culture Collection (ATCC, Manassas, VA, USA). All cell lines were cultured in DMEM supplemented with 10% FBS and 100 U/ml penicillin/streptomycin. Cells were grown to confluence at 37 °C in an atmosphere of 95% humidified air and 5% CO_2_.

### Human tissues

To determine the expression of DIRAS3 in noncancerous tissue, we collected human tongue tissues from surgical resections. Informed patient consent was obtained beforehand. Tongue tissues (No. of samples = 3) were obtained from West China Hospital of Stomatology. Tongue tissues were collected from patients who underwent primary surgical resection of tumors. All human materials were used in accordance with the policies of the institutional review board at West China Hospital of Stomatology.

### Adenovirus cloning and infection

DIRAS3 was amplified by PCR using high-fidelity Taq polymerase (TaKaRa, Dalian, China) and specific primers (forward: 5′-CAGGAATTCATGGGTAACGCCAGCTTTGGC-3′, reverse: 5′-ACGGGATCCTCACATGATTATGCACTTGTC-3′). The PCR products were digested with *Eco*RI and *Bam*HI and ligated into compatible restriction sites of the adenoviral vector pacAd5-CMV-IRES-GFP (Cell Biolabs, San Diego, CA, USA). The adenoviruses were generated by co-transfection of the shuttle vector pacAd5-CMV-IRES-GFP/DIRAS3 and backbone vector pacAd5–9.2-100 into 293A cells using Lipofectamine 2000 reagent (Invitrogen Life Technologies, Carlsbad, CA, USA). The adenovirus titer was increased up to 2 × 10^11^ PFU/ml by cesium chloride gradient ultracentrifugation. The recombinant adenovirus expressing human wild-type p53 was applied by using the first licensed gene therapeutic drug rAd-p53 (Gendicine). For adenovirus infection, CAL-27 and SCC-25 cells were seeded in 6-well plates at a density of 1 × 10^5^ cells/ml and infected with Ad-DIRAS3 and/or rAd-p53 for 24 h. The medium was replaced with normal culture medium, and cells were cultured for 48 h to allow gene expression. Adenovirus infection efficiencies were more than 80%. The Ad-GFP expressing green fluorescent protein was used as control adenovirus.

### Flow cytometric analysis

For cell cycle analysis, cells (1 × 10^5^) were harvested, washed with cold PBS, and pelleted by centrifugation at 1000 r/min for 5 min. Cells were resuspended in 70% ice-cold ethanol overnight at 4 °C. Thereafter, cells were incubated with 50 μg/ml propidium iodide (PI), 100 μg/ml RNAase and 0.1% Triton X-100 for 1 h in the dark. Samples were examined using a FACSCalibur instrument (BD Biosciences, San Jose, CA, USA).

For apoptosis analysis, cells were stained with Annexin V-PE and 7-AAD (PharMingen, San Jose, CA, USA) according to the manufacturer’s instructions. Apoptosis after DIRAS3 and p53 re-expression was evaluated using a FACSCalibur instrument. Cells that were Annexin V^−^/7-AAD^−^ were considered intact, Annexin V^+^/7-AAD^−^ cells were considered early apoptotic, and Annexin V^+^/7-AAD^+^ cells were considered necrotic.

### MTT assay

Cells were incubated with 0.5 mg/ml 3-(4, 5-dimethylthiazol-2-yl)-2, 5-diphenyltetrazolium bromide (MTT) for 4 h at 37 °C. The violet-colored formazan crystals produced by the living cells were dissolved with 100 μl of DMSO. The absorbance of each well was measured at a 570 nm wavelength using a microplate reader (Thermo Fisher Scientific, Rockford, IL, USA).

### Western blotting

Cells and tissues were lysed in RIPA buffer with complete EDTA-free protease inhibitor cocktail (Roche, Mannheim, Germany). Equivalent amounts of protein were subjected to SDS-PAGE and transferred to PVDF membranes. The membranes were probed with specific antibodies. Proteins were visualized with an enhanced chemiluminescence reagent (Millipore, Bedford, MA, USA). Because the anti-LC3 antibodies react preferentially with LC3-II and less so with LC3-I, LC3-II expression levels were normalized to GAPDH rather than LC3-I [15].

### TUNEL assay

Cells cultured on glass coverslips were fixed with 4% paraformaldehyde and then permeabilized with 0.1% Triton X-100 in 0.1% sodium citrate for 2 min on ice. TUNEL reaction mixture (Roche) was added to the coverslips, and the cells were incubated for 1 h at 37 °C. For nuclear staining, cells were incubated with 1 μg/ml DAPI for 15 min at 37 °C. Cells were observed under a fluorescence microscope, and the number of TUNEL-positive cells was counted in 10 randomly selected fields.

### Real-time PCR

Cells and tumor tissues were homogenized in Trizol. One microgram of total RNA was extracted and prepared for cDNA synthesis using the PrimeScript RT reagent kit with gDNA Eraser (Takara). Real-time PCR was carried out using gene-specific primers, cDNAs, SYBR Premix Ex Taq II, and a 7300 Real-Time PCR system (Applied Biosystems, Foster City, CA, USA) according to the manufacturer’s instructions. Relative mRNA levels were calculated using the formula 2^-ΔΔCT^ after normalizing to GAPDH. The primers for each gene are as follows: p53, 5′-GCTTTGAGGTGCGTGTTTGTG-3′ and 5′-ACTTCAGGTGGCTGGAGTGAG-3′; DIRAS3, 5′-TGAGTACCTGCCGACCATTGA-3′ and 5′-ACATCGGTCTTGGCTGAAATC-3′; p21^WAF1/CIP1^, 5′-GACTTTGTCACCGAGACACCAC-3′ and 5′-CTGAGCGAGGCACAAGGGTA-3′; Bax, 5′-GTCGCCCTTTTCTACTTTGCC-3′ and 5′-TTGAGGAGTCTCACCCAACCA-3′; STAT3, 5′-TAACATTCTGGGCACAAACAC-3′ and 5′-TGATACACCTCGGTCTCAAAG-3′; c-Myc, 5′-AATGAAAAGGCCCCCAAGGTAGTTATCC-3′ and 5′-GTCGTTTCCGCAACAAGTCCTCTTC-3′; cyclin D1, 5′-CTGGCCATGAACTACCTGGA-3′ and 5′-GTCACACTTGATCACTCTGG-3′; VEGF, 5′-TGCAGATTATGCGGATCAAACC-3′ and 5′-TGCATTCACATTTGTTGTGCTGTAG-3′; FGF, 5′-GAGAAGAGCGACCCTCACA-3′ and 5′-TAGCTTTCTGCCCAGGTCC-3′; EGFR, 5′-GCGTCTCTTGCCGGAATGT-3′ and 5′-CTTGGCTCACCCTCCAGAAG-3′; Bcl-2, 5′-CGACTTCGCCGAGATGTCCAGCCAG-3′ and 5′-ACTTGTGGCCCAGATAGGCACCCAG-3′; GAPDH, 5′-AACGGGAAGCTTGTCATCAATGGAAA-3′ and 5′-GCATCAGCAGAGGGGGCAGAG-3′.

### Immunoprecipitation

Cells were lysed and incubated with 4E-BP1 antibody overnight at 4 °C. Protein G Sepharose 4 Fast Flow (Sigma-Aldrich) was added, and the solutions were gently mixed. 4E-BP1 was immunoprecipitated from 12,000×*g* supernatants. Samples were subjected to Western blotting using eIF4E antibody to assess the association of 4E-BP1 with eIF4E. The results were normalized to the amount of 4E-BP1.

### Transmission electron microscopy

Cells were harvested, pelleted, and fixed with a solution containing 2.5% glutaraldehyde/2% paraformaldehyde in 0.1 mol/L cacodylate buffer. The samples were postfixed in 2% OsO_4_ for 1 h, dehydrated in a graded series of ethanol, and embedded in Polybed 812. Ultrathin sections (60 nm) were stained with uranyl acetate and lead citrate and photographed under a transmission electron microscope (JEOL, Tokyo, Japan).

### LC3 fluorescence microscopy

Cells were transfected with GFP-LC3 plasmid using Lipofectamine 2000 reagent (Invitrogen Life Technologies). Cells were fixed with 4% paraformaldehyde, washed with PBS, and examined using a fluorescence microscope. The formation of GFP-LC3 puncta was observed, and the number of autophagic cells was calculated in 10 randomly selected fields.

### Murine orthotopic xenografts

Six-week-old BALB/c nu/nu mice were obtained from the Experimental Animal Center of Sichuan University. CAL-27 cells (1 × 10^6^) were intramuscularly injected into the mouth floor as previously reported [16]. Tumor volume was measured every 6 days after injection. When palpable tumors had grown to a diameter of 0.5 cm, the mice were divided into four groups (*n* = 5, each). For adenovirus infection, the mice were intratumorally injected every 3 days with 200 μl of PBS containing Ad-DIRAS3, rAd-p53, Ad-DIRAS3 plus rAd-p53, or control adenovirus. The virus doses for Ad-DIRAS3 and rAd-p53 infection were 1 × 10^9^ PFU/mouse and 1 × 10^10^ VP/kg, respectively. The mice in each group received 4 cycles of adenovirus injection. Animals were sacrificed when the tumor diameter reached approximately 1.0 cm. Major organs (heart, lung, liver, kidney and spleen) were collected, fixed in 4% formalin, and embedded in paraffin. All samples were sectioned into 6 μm slices and subsequently stained with hematoxylin and eosin (H&E). All procedures were carried out according to the animal protocol approved by the Institutional Animal Care and Use Committee of Sichuan University.

### Statistical analysis

Statistical analyses were carried using SPSS 13.0 software (SPSS Inc., Chicago, IL, USA). Statistical analyses were performed using Student’s *t*-test or one-way ANOVA and Tukey’s multiple comparison test. Differences were considered significant with *P*-values < 0.05.

## Results

### Concurrent re-expression of DIRAS3 and p53 decreases proliferation and induces apoptosis and cell cycle arrest in vitro

**Fig. 1:**
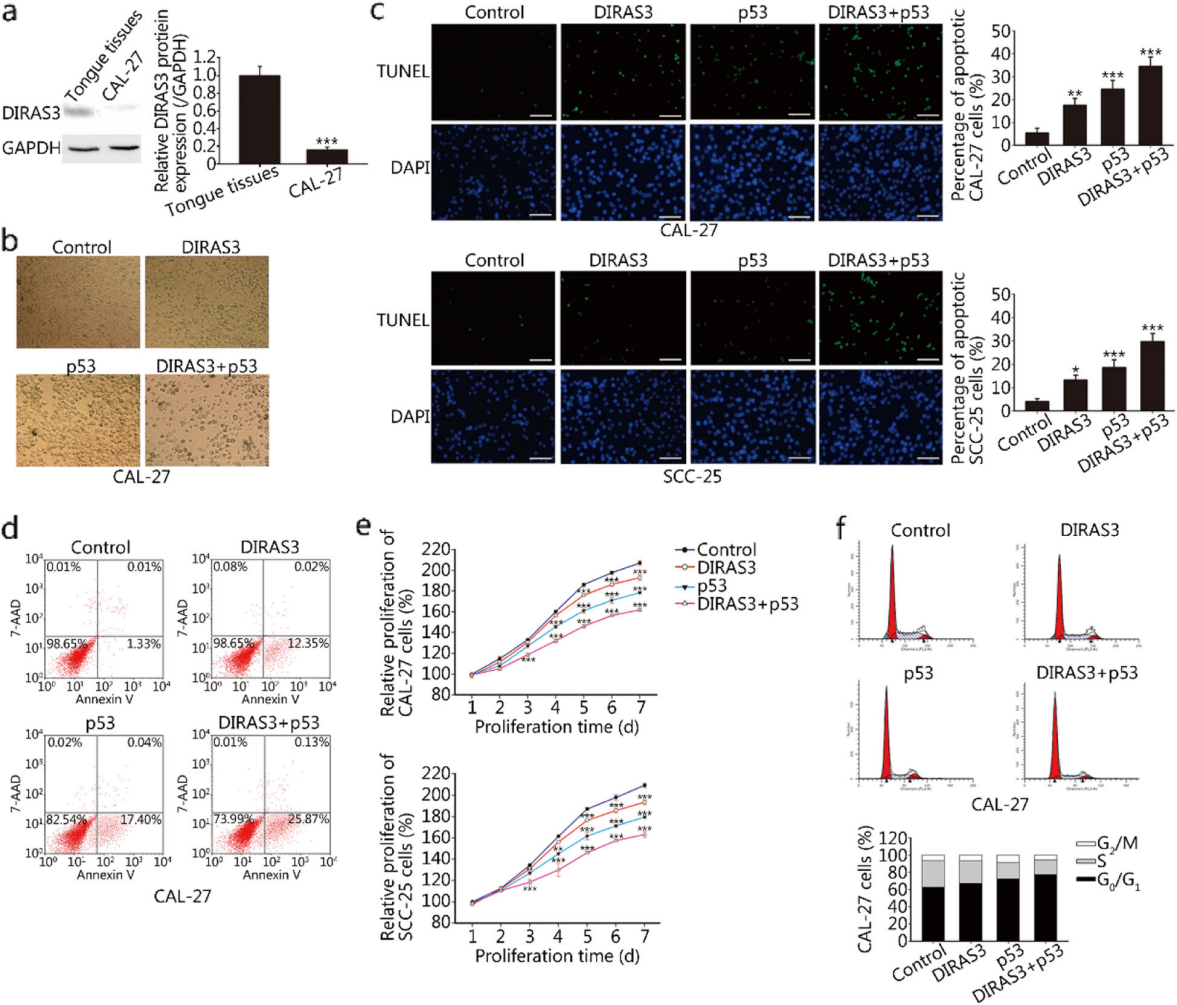
Effects of DIRAS3 and p53 re-expression on the growth and apoptosis of HNSCC cells. **a.** The expression of DIRAS3 protein in CAL-27 HNSCC cells and noncancerous tongue tissues was determined by western blotting. **b.** CAL-27 cells were treated with Ad-DIRAS3, rAd-p53, and their combination for 24 h. Ad-GFP was used as a negative control. The morphologic changes were observed using a bright-field microscope. **c.** Apoptosis after adenoviruses infection was measured by TUNEL assay. The representative images of CAL-27 and SCC-25 cells are shown in the left panels. Green color shown in the images represents positive TUNEL staining, and blue color is from the nuclear dye DAPI. Scale bar, 100 μm. Apoptotic cells in 10 randomly selected fields were counted, and they are shown in the right panels. **d.** The rate of apoptosis was determined by flow cytometric analysis. Cells with Annexin V^+^/7-AAD^−^ staining were considered to be at an early stage of apoptosis. **e.** The effects of adenoviruses on the proliferation of CAL-27 and SCC-25 cells were assessed using MTT after 24 h of infection. **f.** The cell cycle distribution in CAL-27 cells was detected by PI staining and flow cytometric analysis. Representative results from three independent experiments are shown in the upper panels, and the percentage of cells in each phase of the cell cycle is depicted in the lower panels. *P < 0.05; **P < 0.01; ***P < 0.001

Western blotting analysis showed that DIRAS3 was only marginally detected in CAL-27 cells but strongly expressed in normal tongue tissues (Fig. 1a). To address the effects of DIRAS3 and p53 in HNSCC, CAL-27 and SCC-25 cells were treated with Ad-GFP, Ad-DIRAS3, or rAd-p53 alone or with a combination of Ad-DIRAS3 and rAd-p53. Cells treated with Ad-GFP or Ad-DIRAS3 with GFP-tagged reporter constructs exhibited green fluorescence (Additional file 1: Fig. S1). Observation using a bright-field microscope showed that the concurrent re-expression of DIRAS3 and p53 in CAL-27 cells reduced cell density (Fig. 1b). TUNEL assay showed that the re-expression of either DIRAS3 or p53 alone induced significant apoptosis in CAL-27 and SCC-25 cells. However, the maximal incidence of TUNEL^+^ cells was observed after the concurrent re-expression of DIRAS3 and p53 (Fig. 1c). Flow cytometric analysis was utilized for quantitative analysis of the number of apoptotic cells. Significant increases in early apoptotic cells (Annexin V^+^/7-AAD^−^) were detected in Ad-DIRAS3 (12.35%), rAd-p53 (17.40%) and the combination group (25.87%) compared with the control group (1.33%) (Fig. 1d). MTT assay showed that the induction of DIRAS3 or p53 individually decreased proliferation in CAL-27 and SCC-25 cells and that the combination treatment resulted in the maximal loss of proliferation capacity (Fig. 1e). Moreover, DIRAS3 in combination with p53 could induce G_0_/G_1_ cell cycle arrest, as shown by the accumulation of cells in G_0_/G_1_ phase with a concomitant decrease in S phase (Fig. 1f).

### DIRAS3 and p53 re-expression modulates downstream genes involved in apoptosis, proliferation and cell cycle arrest

**Fig. 2:**
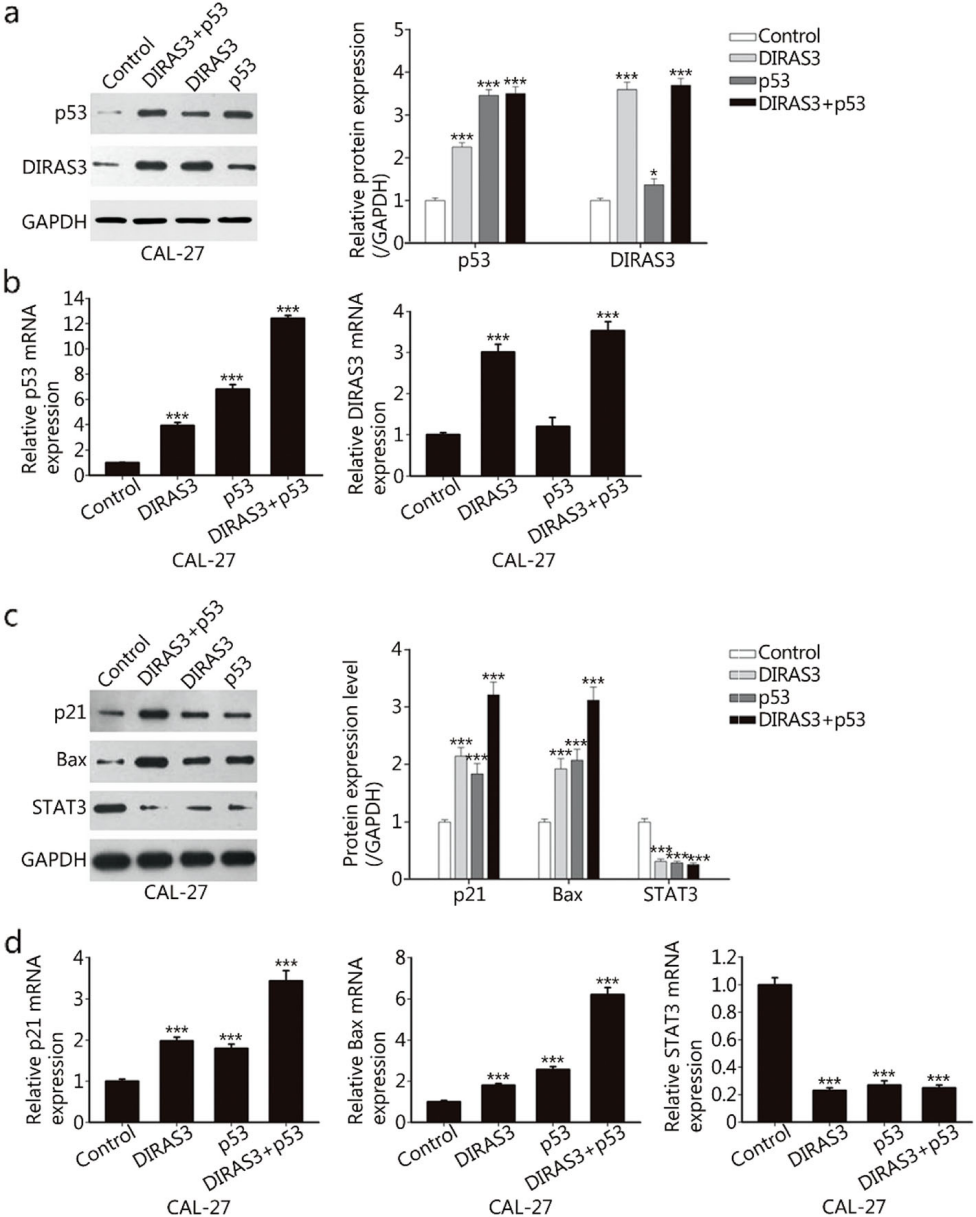
DIRAS3 and p53 re-expression modulates downstream genes involved in apoptosis, proliferation and cell cycle arrest. **a.** CAL-27 cells were treated with Ad-DIRAS3, rAd-p53, and their combination. Ad-GFP was used as a negative control. After 24 h of infection, whole cell lysates were collected, and the expression of DIRAS3 and p53 was detected by western blotting. **b.** Relative mRNA expression levels of DIRAS3 and p53 were determined by real-time PCR and calculated by normalizing to GAPDH. The fold changes are relative to the control group, to which a value of 1.0 was assigned. **c.** The expression of the three genes p21^WAF1/CIP1^, Bax and STAT3 was examined by Western blotting after adenovirus infection. **d.** Relative mRNA levels of p21^WAF1/CIP1^, Bax and STAT3 were detected by real-time PCR. ***P < 0.001

Western blotting analysis showed that Ad-DIRAS3 significantly upregulated p53. Conversely, only a slight increase in DIRAS3 expression was observed in response to rAd-p53 infection (Fig. 2a). Real-time PCR demonstrated that Ad-DIRAS3 significantly increased the p53 mRNA level; however, rAd-p53 failed to significantly increase DIRAS3 mRNA. The combination treatment resulted in the largest increases in DIRAS3 and p53 mRNA levels compared with that of any single agent treatment (Fig. 2b).

The expression of three genes downstream of DIRAS3 and p53 were examined. We observed that the concurrent re-expression of DIRAS3 and p53 significantly induced the expression of p21^WAF1/CIP1^ and Bax compared to the treatment of cells with DIRAS3 or p53 individually (Fig. 2c). However, the expression of STAT3 did not further decrease in the combination treatment group compared with that in the DIRAS3 or p53 alone group (Fig. 2c). Real-time PCR also showed that compared with the treatment of cells with any single agent, the combination treatment induced more significant changes in the expression of p21^WAF1/CIP1^ and Bax, while it did not exhibit any synergistic effects on the reduction in STAT3 (Fig. 2d).

### DIRAS3 and p53 re-expression increases the interaction of 4E-BP1 with eIF4E via the regulation of Akt

**Fig. 3:**
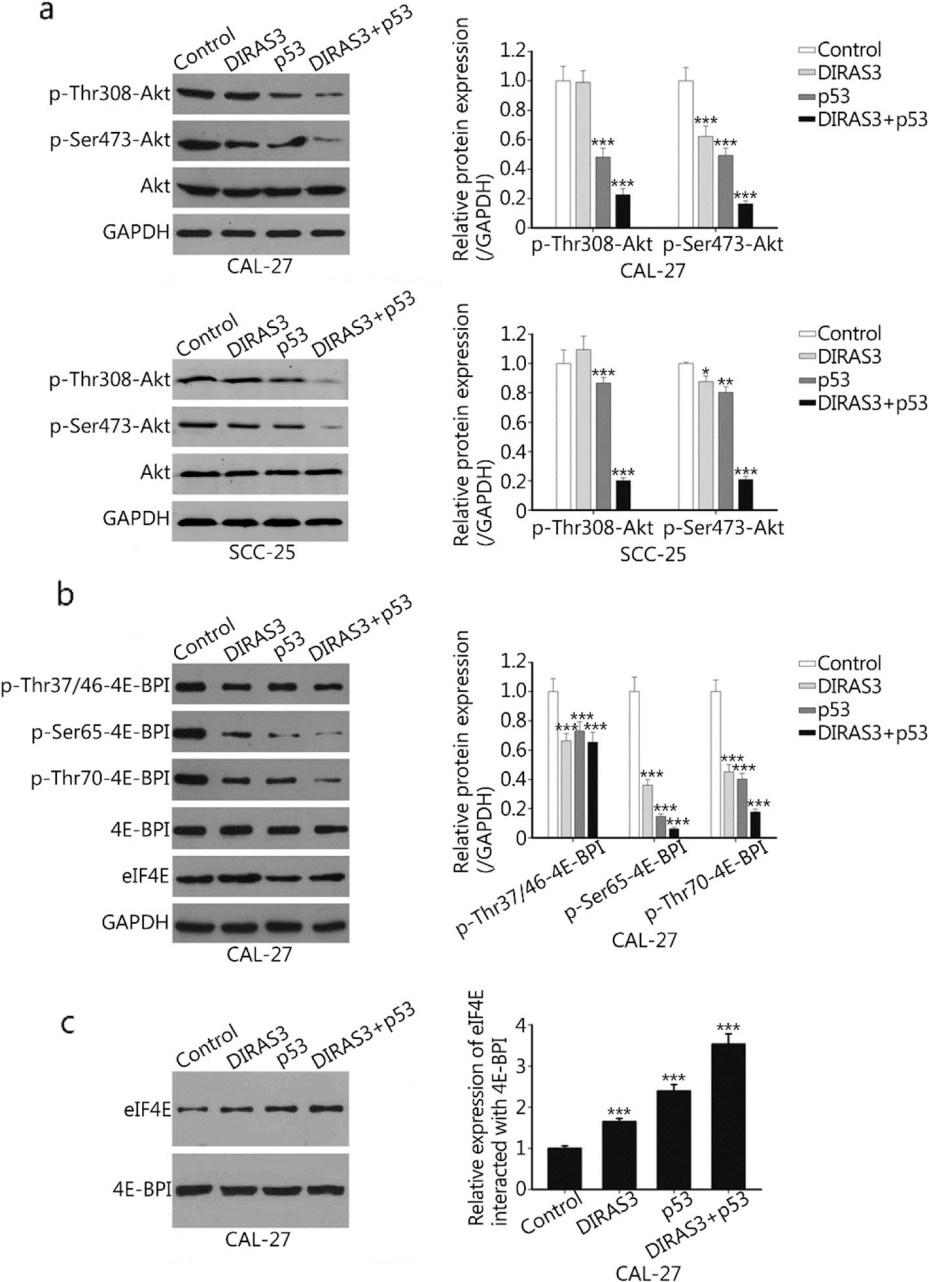
Effects of DIRAS3 and p53 re-expression on the interaction of 4E-BP1 with eIF4E. **a**, **b**. Cancer cells were treated with Ad-DIRAS3, rAd-p53, and their combination for 24 h. Ad-GFP was used as a negative control. Whole cell lysates were collected and subjected to western blotting. The expression of Akt, p-Thr308-Akt, and p-Ser473-Akt in CAL-27 and SCC-25 cells was examined (**a**). The levels of 4E-BP1, p-Thr37/46-4E-BP1, p-Ser65-4E-BP1, p-Thr70-4E-BP1, and eIF4E in CAL-27 cells were also analyzed (**b**). **c.** Cell extracts were immunoprecipitated with an anti-4E-BP1 antibody. The interaction of 4E-BP1 with eIF4E in CAL-27 cells was evaluated by measuring the amount of eIF4E in the immunoprecipitates by Western blotting. 4E-BP1 was used as a loading control

Western blotting analysis showed that the re-expression of DIRAS3 suppressed the phosphorylation of Akt at Ser473 but not at Thr308, while p53 inhibited Akt phosphorylation at both Thr308 and Ser473. The concurrent re-expression of DIRAS3 and p53 nearly abolished the activity of Akt, as evidenced by the significantly decreased Akt phosphorylation at both residues (Fig. 3a).

4E-BP1 is a key downstream target of the PI3K/Akt pathway. As shown in Fig. 3b, DIRAS3 or p53 induced hypo-phosphorylation of 4E-BP1. The phosphorylation of 4E-BP1 at Ser65, Thr70 and Thr37/46 was significantly inhibited by DIRAS3 or p53 re-expression. DIRAS3 in combination with p53 further reduced the phosphorylation at Ser65 and Thr70, while there was no further change in the phosphorylation of Thr37/46. Moreover, increased interaction of 4E-BP1 with eIF4E was observed in the DIRAS3 or p53 group, but the maximal amount of eIF4E bound to 4E-BP1 was observed in the combination treatment group (Fig. 3c).

### DIRAS3 and p53 re-expression inhibits the expression of multiple oncogenes

**Fig. 4:**
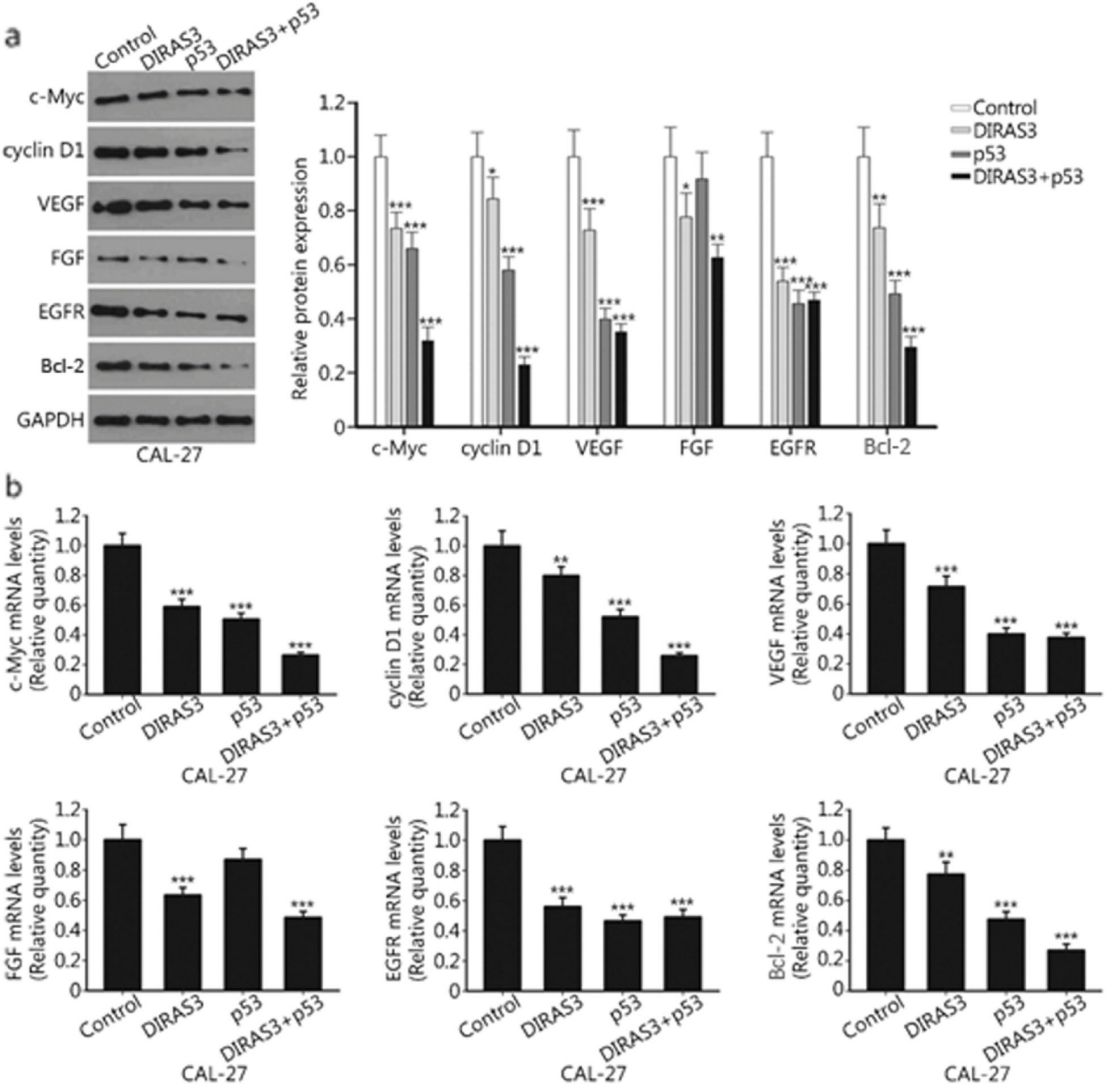
DIRAS3 and p53 re-expression inhibits the expression of multiple oncogenes in HNSCC cells. **a.** CAL-27 cells were treated with Ad-DIRAS3, rAd-p53, and their combination for 24 h. Ad-GFP was used as a negative control. The expression of c-Myc, cyclin D1, VEGF, FGF, EGFR and Bcl-2 proteins was determined by western blotting. **b.** Relative mRNA levels of c-Myc, cyclin D1, VEGF, FGF, EGFR and Bcl-2 were examined by real-time PCR. **P < 0.01; ***P < 0.001

Western blotting analysis showed that cell cycle regulators c-Myc and cyclin D1 were downregulated after DIRAS3 or p53 re-expression. The expression of angiogenic factors VEGF, EGFR, as well as pro-survival protein Bcl-2, was also inhibited by either single agent. FGF was downregulated after DIRAS3 re-expression. Moreover, the combination treatment substantially suppressed the expression of c-Myc, cyclin D1, FGF and Bcl-2. By contrast, the expression of VEGF and EGFR could not be further decreased in the combination treatment group compared with that in the p53 group (Fig. 4a). The real-time PCR results showed similar expression patterns of these oncogenes. The concurrent re-expression of DIRAS3 and p53 significantly reduced the expression of c-Myc, cyclin D1, VEGF, FGF, EGFR and Bcl-2 (Fig. 4b).

### DIRAS3 and p53 re-expression increases the accumulation of autophagosomal LC3 and induces impaired autophagy

**Fig. 5:**
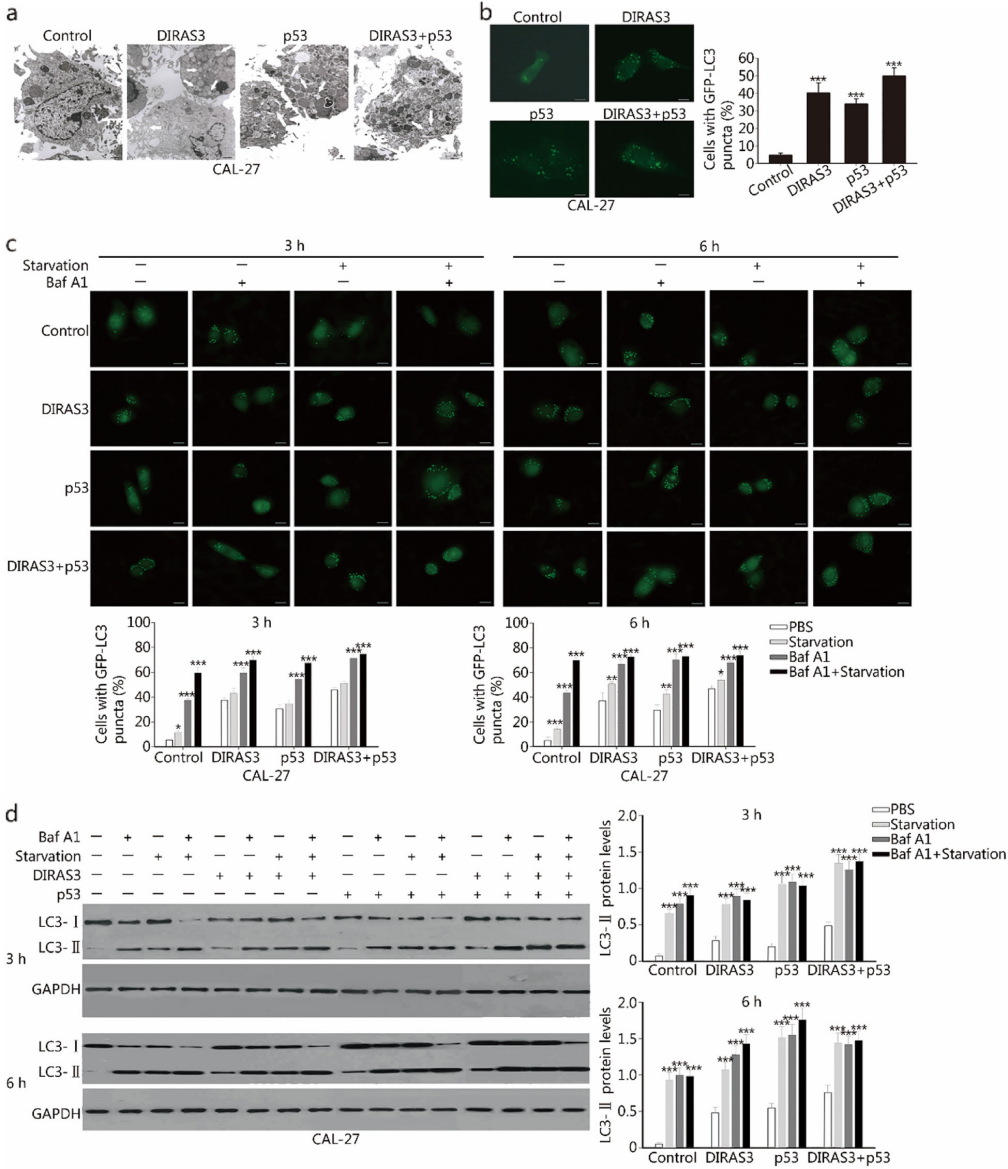
DIRAS3 and p53 re-expression stimulates autophagy in HNSCC cells. **a.** CAL-27 cells were treated with Ad-DIRAS3, rAd-p53, and their combination for 24 h. Ad-GFP was used as a negative control. Autophagy was determined by examining the cellular AVs using transmission electron microscopy. Representative images of cells that developed AVs are shown. White arrows indicate AVs. Scale bar, 5 μm. **b.** The formation of GFP-LC3 puncta was observed using fluorescence microscope. The percentage of cells with GFP-LC3 puncta in each group was as follow: Control group: 4.67% ± 1.25%, DIRAS3 group: 40.33% ± 5.73%, p53 group: 34.00% ± 2.94%, DIRAS3 plus p53 group: 50.00% ± 4.55%. Representative images and the quantification of GFP-LC3 puncta are shown. Scale bar, 20 μm. **c.** CAL-27 cells were treated with Ad-GFP, Ad-DIRAS3, rAd-p53, and Ad-DIRAS3 plus rAd-p53. After 24 h of infection, cells were further starved in EBSS or treated with 100 nM Baf A1 for 3 h or 6 h. The formation of GFP-LC3 puncta was observed using fluorescence microscope. Representative images and the quantification of GFP-LC3 puncta are shown. The autophagic flux was deduced from the ratio between the percentage of cells with GFP-LC3 puncta in the presence of Baf A1 and the percentage in the presence of PBS [17]. Scale bar, 20 μm. **d.** CAL-27 cells were treated as in (**c**). The levels of LC3-I and LC3-II were detected by western blotting. The expression of LC3-II was calculated after normalizing to GAPDH. The autophagic flux was deduced from the LC3-II expression levels in the presence of Baf A1 compared to the levels in the presence of PBS [17]. *P < 0.05; **P < 0.01; ***P < 0.001

A significantly increased accumulation of autophagic vacuoles (AVs) was observed in cells re-expressed with DIRAS3, p53, and the combination as compared with that in the control adenovirus-treated cells, which suggested that DIRAS3 and p53 could induce autophagy in CAL-27 cells (Fig. 5a). The formation of punctate dots with GFP-LC3 fusion protein is a well-characterized feature to visualize autophagosomes [18]. We found that the percentage of cells with GFP-LC3 puncta was increased after DIRAS3 or p53 re-expression. The combination treatment induced the maximum percentage of cells with punctate LC3 dots (Fig. 5b).

Next, cells transfected with adenoviruses were either starved in Earle’s balanced salt solution (EBSS) or treated with Baf A1. We observed that these cells were fully competent after starvation or treatment with Baf A1 (3 h and 6 h starvation or Baf A1 treatment). In the presence of Baf A1, DIRAS3 and p53 re-expression continued to induce GFP-LC3 puncta, indicating that the combination treatment could increase autophagic activity in CAL-27 cells (Fig. 5c). We also evaluated the autophagic flux and found that the autophagic flux in the combination treatment group (25.3% in the 3 h group and 21.0% in the 6 h group) was reduced compared with that in cells treated with control adenovirus (32.0% in the 3 h group and 38.7% in the 6 h group, Fig. 5c). Western blotting analysis showed that the simultaneous re-expression of DIRAS3 and p53 increased LC3-II expression in Baf A1-treated cells (Fig. 5d). The autophagic flux in the combination treatment group (0.66 in 6 h group) was lower than that in cells treated with control adenovirus (0.95 in 6 h group, Fig. 5d). LC3-II levels accumulated copiously after the combination treatment in starved cells (Fig. 5d).

### DIRAS3 and p53 re-expression inhibits tumor growth in HNSCC xenograft models

**Fig. 6:**
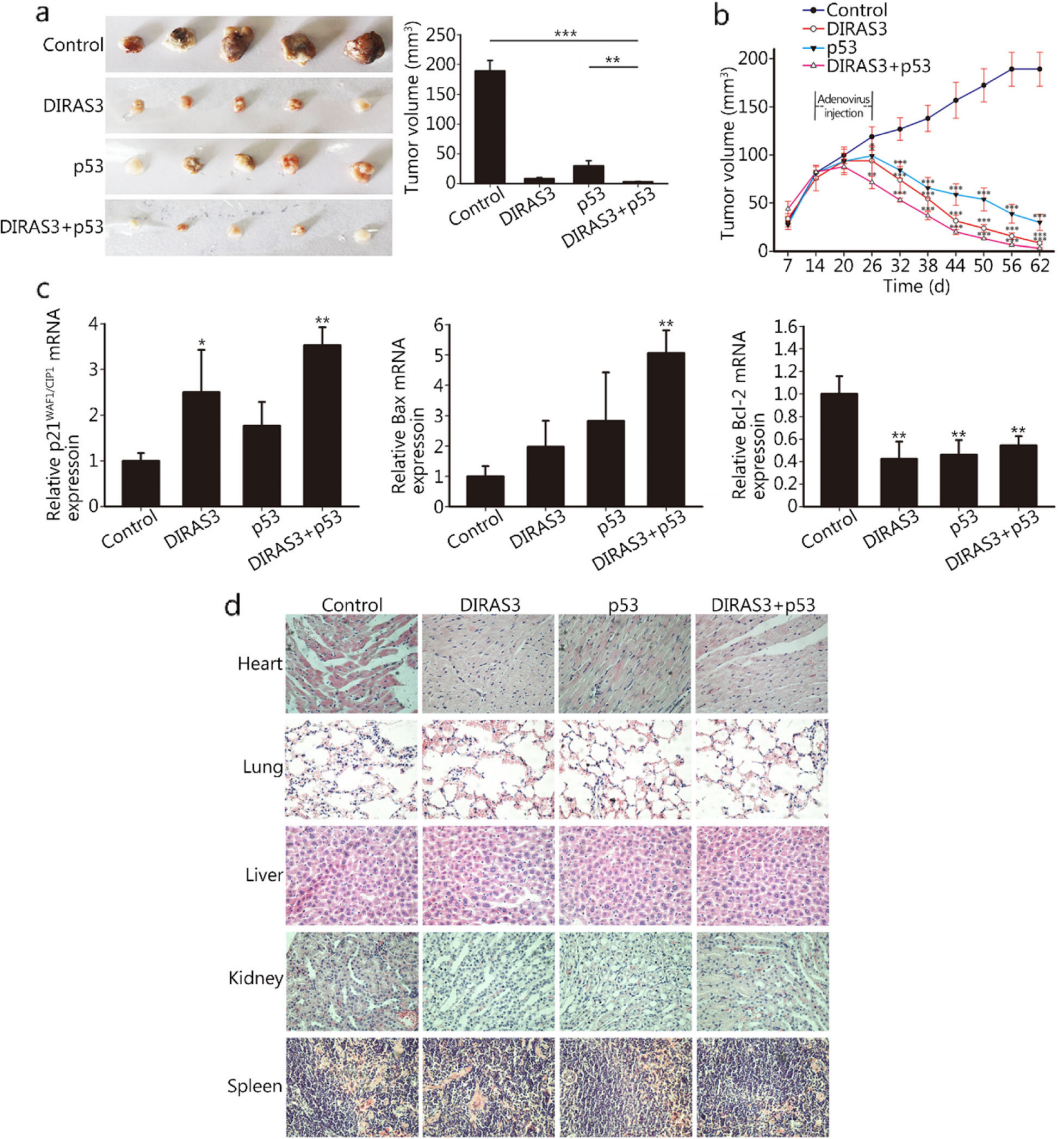
Effects of DIRAS3 and p53 re-expression on tumor growth in vivo. CAL-27 xenograft-bearing nude mice were intratumorally injected every 3 days with Ad-GFP, Ad-DIRAS3, rAd-p53, or Ad-DIRAS3 plus rAd-p53 (No. of mice for each group = 5). Nude mice were treated with 4 cycles of adenovirus injection in 12 days. Tumor diameters were measured every 6 days, and the volume was calculated using the following formula: π/6 × larger diameter×(smaller diameter)^2^. At 36 days after the end of adenovirus injection, mice were sacrificed, and tumors were excised. Each tumor in four groups was shown, and the tumor volume was calculated as shown in (**a**). The tumor volume at the endpoints of the experiment in each group was as follow: Control group: 189.02 ± 17.54 mm^3^, DIRAS3 group: 8.45 ± 1.73 mm^3^, p53 group: 29.99 ± 8.38 mm^3^, DIRAS3 plus p53 group: 3.12 ± 0.75 mm^3^. The tumor volume at different time points was plotted in (**b**). Relative mRNA levels of p21^WAF1/CIP1^, Bax and Bcl-2 in tumor tissue homogenates were examined by real-time PCR (**c**). Heart, lung, liver, kidney and spleen sections were stained with hematoxylin and eosin (H&E) for histopathological analysis (**d**). *P < 0.05; **P < 0.01; ***P < 0.001

The antitumoral effects of DIRAS3 and p53 were validated in a murine orthotopic xenograft model. Ad-DIRAS3 injection significantly suppressed tumor growth compared with that in mice treated with control adenovirus. A weaker inhibition of tumor growth was observed after rAd-p53 injection. The combination treatment resulted in the largest inhibition rate of tumor growth. At the endpoints of the experiment, the combination treatment induced significant inhibition of xenograft tumor growth compared with control adenovirus or rAd-p53. The tumor volume in the combination treatment group was slightly smaller than that in the Ad-DIRAS3 alone group, but the difference did not reach statistical significance (Fig. 6a, b). Next, we analyzed the expression of p21^WAF1/CIP1^, Bax and Bcl-2 from isolated tumors by real-time PCR. Our results showed that the combination treatment significantly upregulated p21^WAF1/CIP1^ and Bax and downregulated Bcl-2 in vivo (Fig. 6c), which was in accordance with our in vitro results. Moreover, H&E staining of different organs (heart, lung, liver, kidney and spleen) showed that there were no obvious histopathological lesions in mice treated with Ad-DIRAS3, rAd-p53 or Ad-DIRAS3 plus rAd-p53 compared with those treated with control adenovirus (Fig. 6d).

## Discussion

In this study, we demonstrated that DIRAS3 expression was suppressed in CAL-27 HNSCC cells. Since DIRAS3 and p53 are both tumor suppressor genes that are frequently mutated or inactivated in malignancies, including HNSCC [10, 19], we first re-expressed DIRAS3 and p53 in CAL-27 and SCC-25 cells using an adenoviral vector expressing DIRAS3 and Gendicine, a recombinant adenovirus p53 agent, respectively. Our data indicated that compared to the treatment of cells with either control adenovirus or a single adenovirus, the combined gene therapy of DIRAS3 and p53 resulted in decreases in proliferation, the induction of G_0_/G_1_ cell cycle arrest, increases in apoptosis, and impaired autophagy. In agreement with the observations in vitro, the concurrent re-expression of DIRAS3 and p53 induced a marked reduction in CAL-27 xenograft tumor growth in vivo. These results suggest that the concurrent targeting of DIRAS3 and p53 represents a more effective approach for the treatment of HNSCC than the targeting of a single gene.

HNSCC develops as a result of multiple genetic alterations; hence, targeting a single gene produces only a modest clinical benefit. Moreover, recent studies suggest that p53 activity is regulated not only by its mRNA expression but also by its nuclear localization, post-translational modifications, and short half-life [20]. For example, MDM2 could bind and ligate ubiquitin to p53, targeting it for rapid degradation by the 26S proteasome [21]. Therefore, a rational approach for the effective inhibition of tumor growth would be to combine Gendicine with multiple agents targeting different tumor suppressor genes. In this study, we found that p53 was dramatically upregulated by Ad-DIRAS3 infection and that the concurrent re-expression of DIRAS3 and p53 induced an even higher level of p53 expression. These results are in agreement with those of previous reports that showed that the re-expression of DIRAS3 increased p53 protein in human pancreatic carcinoma cells [22]. Akt is reported to enhance MDM2-mediated ubiquitination and degradation of p53 [23], and Akt activity was significantly inhibited after DIRAS3 re-expression in our study. These results suggest that DIRAS3 upregulates p53 at least in part via Akt inactivation.

p21^WAF1/CIP1^, Bax and STAT3 are important genes involved in proliferation and oncogenesis. p21^WAF1/CIP1^ is an inhibitor of cyclin-dependent kinase and is correlated with the prognosis in head and neck cancer [24, 25]. Bax is directly activated by p53, allowing for further mitochondrial membrane permeabilization and apoptosis [26, 27]. Overexpression of Bax enhanced the anticancer activity of multiple chemotherapeutic agents in head and neck cancer [28]. STAT3 activation not only promotes cell growth and survival but also induces resistance to conventional therapies that rely on apoptotic machinery to eliminate cancer cells [29]. In our study, the combination treatment showed synergistic effects on the modulation of p21^WAF1/CIP1^ and Bax. Therefore, the combination treatment of DIRAS3 and p53 was far more effective for inhibiting HNSCC tumor growth than either single agent.

The primary functions of Akt include the promotion of cell survival, proliferation, metabolism, and angiogenesis in response to various stimuli [30]. Recently, Akt was reported to play an important role in the regulation of 4E-BP1 phosphorylation, thereby controlling the mRNA translation of IFN- and insulin-stimulated genes [31, 32]. We showed that the concurrent re-expression of DIRAS3 and p53 significantly inhibited the phosphorylation of Akt and 4E-BP1. eIF4E recognizes the 5′-cap structure of mRNAs, delivering these mRNAs to the eIF4F complex for translation initiation. However, 4E-BP1 could bind eIF4E, thereby preventing eIF4F complex assembly and translation initiation [33, 34]. The repression of eIF4E function preferentially and disproportionately reduces the expression of numerous growth and survival factors critical for malignancy [35]. The genetic silencing of eIF4E, or pharmacologic inhibition using ribavirin, reduces the growth, invasion and metastasis of breast cancer [36]. eIF4E silencing also increases Bax/Bcl-2 ratio and sensitizes breast cancer to cisplatin, adriamycin, paclitaxel and docetaxel [37]. In our study, the concurrent re-expression of DIRAS3 and p53 significantly promoted the binding of 4E-BP1 to eIF4E. Moreover, the expression of eIF4E-regulated oncogenes (e.g., c-Myc, cyclin D1, VEGF, FGF, EGFR, Bcl-2) was inhibited after DIRAS3 and p53 re-expression. From this data, we conclude that the suppression of eIF4E function is partly responsible for the antitumor effects of DIRAS3 and p53.

Autophagy is a catabolic process that delivers cytoplasmic components such as long-lived proteins and senescent organelles to lysosomes for degradation. Autophagy is regarded as a survival mechanism under adverse conditions to provide energy or maintain cell integrity. However, excessive autophagy leads to autophagic (type II) cell death [38]. DIRAS3 is essential for the induction of autophagy [39]. Its overexpression in epithelial ovarian cancer cells induces apoptosis and autophagic cell death via inhibition of PI3K/Akt and Bcl-2 [8]. p53 binds to the promoter region of multiple genes that code for pro-autophagic regulators, including AMPK β_1_ and β_2_ subunits, DAPK-1, DRAM, pro-apoptotic Bcl-2 proteins (e.g. Bad, Bax, BNIP3, and Puma), Sestrin-2, and TSC2 [40, 41]. In the current study, we showed that the concurrent re-expression of DIRAS3 and p53 induced autophagy, as evidenced by the increased formation of AVs and GFP-LC3 puncta, and enhanced LC3-II conversion in HNSCC cells.

The increased formation of AVs and GFP-LC3 puncta may be explained by increased autophagosome formation due to the induction of autophagic activity or the inhibition of autophagosome clearance due to the decreased flux of autophagosomes to autolysosomes [42]. We analyzed whether the concurrent re-expression of DIRAS3 and p53 could regulate different stages of autophagy. We found that the combination treatment significantly inhibited the phosphorylation of Akt, suggesting that it stimulates early signaling to enhance the initiation of autophagy. Indeed, the re-expression of DIRAS3 and p53 was effective to induce the formation of GFP-LC3 puncta in the case of stalled autophagic flux by Baf A1. In addition to enhance the formation of autophagosomes, our study also suggests an inhibitory effect of DIRAS3 and p53 re-expression on the late stage of autophagy. This conclusion is derived from 2 lines of evidence. First, the concurrent re-expression of DIRAS3 and p53 inhibited autophagic flux in HNSCC cells. Second, the combination treatment had a larger effect on the starvation-induced production of GFP-LC3 puncta and the conversion of LC3-I to LC3-II. Through the regulation of both the early and late stages of autophagy, the combination treatment impaired autophagy. Autophagy can facilitate survival by promoting adaptation to adverse environments or can induce cell death due to excessive removal of mitochondria. The re-expression of DIRAS3 was reported to induce autophagic cell death in ovarian and breast cancer cells [9, 43]. Crighton et al. showed that p53 induces autophagy in a DRAM-dependent manner, and DRAM is essential for p53-mediated apoptosis [44]. However, another study showed that p53 re-activation by CP-31398 and RITA induces protective autophagy [45]. It is unclear whether the impaired autophagy induced by DIRAS3 and p53 re-expression in our study plays a pro-survival or pro-apoptotic role. Therefore, the effects of impaired autophagy by DIRAS3 and p53 re-expression on cancer cell death need further investigation.

## Conclusions

Overall, our study demonstrates that the concurrent re-expression of DIRAS3 and p53 induces multiple anti-tumor effects and suggests a more effective approach for the treatment of HNSCC than the currently used treatment strategies.

## Supplementary information

**Additional file 1: Fig. S1** Expression of GFP in cells treated with Ad-GFP, Ad-DIRAS3, or rAd-p53 alone or with a combination of Ad-DIRAS3 and rAd-p53.

## Data Availability

Not applicable.

## Abbreviations

4E-BP1: Eukaryotic initiation factor 4E-binding protein 1
DIRAS3: DIRAS family GTPase 3
AVs: Autophagic vacuoles
EGFR: Epidermal growth factor receptor
eIF4E: Eukaryotic initiation factor 4E
FGF: Fibroblast growth factor
HNSCC: Head and neck squamous cell carcinoma
LC3: Microtubule-associated protein light chain 3
VEGF: Vascular endothelial growth factor

## References

[1] McDermott JD, Bowles DW (2019). Epidemiology of head and neck squamous cell carcinomas: impact on staging and prevention strategies. Curr Treat Options in Oncol.

[2] Leemans CR, Braakhuis BJ, Brakenhoff RH (2011). The molecular biology of head and neck cancer. Nat Rev Cancer.

[3] Tassone P, Old M, Teknos TN, Pan Q (2013). p53-based therapeutics for head and neck squamous cell carcinoma. Oral Oncol.

[4] Philips R, Pan Q, Warnakulasuriya S, Khan Z (2017). p53 in head and neck squamous cell carcinoma. Squamous cell carcinoma.

[5] Lane DP, Cheok CF, Lain S (2010). p53-based cancer therapy. Cold Spring Harb Perspect Biol.

[6] Zhang WW, Li L, Li D, Liu J, Li X, Li W (2018). The first approved gene therapy product for cancer ad-p53 (Gendicine): 12 years in the clinic. Hum Gene Ther.

[7] Ma G, Shimada H, Hiroshima K, Tada Y, Suzuki N, Tagawa M (2008). Gene medicine for cancer treatment: commercially available medicine and accumulated clinical data in China. Drug Des Devel Ther.

[8] Li J, Cui G, Sun L, Wang SJ, Tian S, Guan Z (2014). ARHI overexpression induces epithelial ovarian cancer cell apoptosis and excessive autophagy. Int J Gynecol Cancer.

[9] Zou CF, Jia LQ, Jin HY, Yao M, Zhao NQ, Huan J (2011). Re-expression of ARHI (DIRAS3) induces autophagy in breast cancer cells and enhances the inhibitory effect of paclitaxel. BMC Cancer.

[10] Zhang S, Feng XL, Shi L, Gong CJ, He ZJ, Wu HJ (2013). Genome-wide analysis of DNA methylation in tongue squamous cell carcinoma. Oncol Rep.

[11] Sutton MN, Lu Z, Li YC, Zhou Y, Huang T, Reger AS (2019). DIRAS3 (ARHI) blocks RAS/MAPK signaling by binding directly to RAS and disrupting RAS clusters. Cell Rep.

[12] Zhao X, Li J, Zhuo J, Cai L (2010). Reexpression of ARHI inhibits tumor growth and angiogenesis and impairs the mTOR/VEGF pathway in hepatocellular carcinoma. Biochem Biophys Res Commun.

[13] Badgwell DB, Lu Z, Le K, Gao F, Yang M, Suh GK (2012). The tumor suppressor gene ARHI (DIRAS3) suppresses ovarian cancer cell migration through inhibition of the Stat3 and FAK/rho signaling pathways. Oncogene..

[14] Li Y, Li B, Li CJ, Li LJ (2015). Key points of basic theories and clinical practice in rAd-p53 (Gendicine ™) gene therapy for solid malignant tumors. Expert Opin Biol Ther.

[15] Mizushima N, Yoshimori T (2007). How to interpret LC3 immunoblotting. Autophagy..

[16] Bais MV, Kukuruzinska M, Trackman PC (2015). Orthotopic non-metastatic and metastatic oral cancer mouse models. Oral Oncol.

[17] Scherz-Shouval R, Weidberg H, Gonen C, Wilder S, Elazar Z, Orena M (2010). p53-dependent regulation of autophagy protein LC3 supports cancer cell survival under prolonged starvation. Proc Natl Acad Sci U S A.

[18] Tanida I, Ueno T, Kominami E (2008). LC3 and autophagy. Methods Mol Biol.

[19] Nichols AC, Yoo J, Palma DA, Fung K, Franklin JH, Koropatnick J (2012). Frequent mutations in TP53 and CDKN2A found by next-generation sequencing of head and neck cancer cell lines. Arch Otolaryngol Head Neck Surg.

[20] Bykov VJN, Eriksson SE, Bianchi J, Wiman KG (2018). Targeting mutant p53 for efficient cancer therapy. Nat Rev Cancer.

[21] Mohammad RM, Wu J, Azmi AS, Aboukameel A, Sosin A, Wu S (2009). An MDM2 antagonist (MI-319) restores p53 functions and increases the life span of orally treated follicular lymphoma bearing animals. Mol Cancer.

[22] Lu X, Qian J, Yu Y, Yang H, Li J (2009). Expression of the tumor suppressor ARHI inhibits the growth of pancreatic cancer cells by inducing G1 cell cycle arrest. Oncol Rep.

[23] Abraham AG, O'Neill E (2014). PI3K/Akt-mediated regulation of p53 in cancer. Biochem Soc Trans.

[24] Llanos S, García-Pedrero JM, Morgado-Palacin L, Rodrigo JP, Serrano M (2016). Stabilization of p21 by mTORC1/4E-BP1 predicts clinical outcome of head and neck cancers. Nat Commun.

[25] Shamloo B, Usluer S (2019). p21 in cancer research. Cancers..

[26] Dashzeveg N, Yoshida K (2015). Cell death decision by p53 via control of the mitochondrial membrane. Cancer Lett.

[27] Zhang H, Feng YW, Yao YM (2018). Potential therapy strategy: targeting mitochondrial dysfunction in sepsis. Military Med Res.

[28] Guo B, Cao S, Tóth K, Azrak RG, Rustum YM (2000). Overexpression of Bax enhances antitumor activity of chemotherapeutic agents in human head and neck squamous cell carcinoma. Clin Cancer Res.

[29] Yin ZJ, Jin FG, Liu TG, Fu EQ, Xie YH, Sun RL (2011). Overexpression of STAT3 potentiates growth, survival, and radioresistance of non-small-cell lung cancer (NSCLC) cells. J Surg Res.

[30] Martini M, De Santis MC, Braccini L, Gulluni F, Hirsch E (2014). PI3K/AKT signaling pathway and cancer: an updated review. Ann Med.

[31] Kaur S, Sassano A, Dolniak B, Joshi S, Majchrzak-Kita B, Baker DP (2008). Role of the Akt pathway in mRNA translation of interferon-stimulated genes. Proc Natl Acad Sci U S A.

[32] Takata M, Ogawa W, Kitamura T, Hino Y, Kuroda S, Kotani K (1999). Requirement for Akt (protein kinase B) in insulin-induced activation of glycogen synthase and phosphorylation of 4E-BP1 (PHAS-1). J Biol Chem.

[33] Wang Z, Feng X, Molinolo AA, Martin D, Vitale-Cross L, Nohata N (2019). 4E-BP1 is a tumor suppressor protein reactivated by mTOR inhibition in head and neck cancer. Cancer Res.

[34] Chen X, Kopecky DJ, Mihalic J, Jeffries S, Min X, Heath J (2012). Structure-guided design, synthesis, and evaluation of guanine-derived inhibitors of the eIF4E mRNA-cap interaction. J Med Chem.

[35] Musa J, Orth MF, Dallmayer M, Baldauf M, Pardo C, Rotblat B (2016). Eukaryotic initiation factor 4E-binding protein 1 (4E-BP1): a master regulator of mRNA translation involved in tumorigenesis. Oncogene..

[36] Pettersson F, Del Rincon SV, Emond A, Huor B, Ngan E, Ng J (2015). Genetic and pharmacologic inhibition of eIF4E reduces breast cancer cell migration, invasion, and metastasis. Cancer Res.

[37] Zhou FF, Yan M, Guo GF, Wang F, Qiu HJ, Zheng FM (2011). Knockdown of eIF4E suppresses cell growth and migration, enhances chemosensitivity and correlates with increase in Bax/Bcl-2 ratio in triple-negative breast cancer cells. Med Oncol.

[38] Yang Y, Jiang G, Zhang P, Fan J (2015). Programmed cell death and its role in inflammation. Military Med Res.

[39] Lu Z, Luo RZ, Lu Y, Zhang X, Yu Q, Khare S (2008). The tumor suppressor gene ARHI regulates autophagy and tumor dormancy in human ovarian cancer cells. J Clin Invest.

[40] Maiuri MC, Galluzzi L, Morselli E, Kepp O, Malik SA, Kroemer G (2010). Autophagy regulation by p53. Curr Opin Cell Biol.

[41] Bieging KT, Mello SS, Attardi LD (2014). Unravelling mechanisms of p53-mediated tumour suppression. Nat Rev Cancer.

[42] Bae EJ, Lee HJ, Jang YH, Michael S, Masliah E, Min DS (2014). Phospholipase D1 regulates autophagic flux and clearance of α-synuclein aggregates. Cell Death Differ.

[43] Washington MN, Suh G, Orozco AF, Sutton MN, Yang H, Wang Y (2015). ARHI (DIRAS3)-mediated autophagy-associated cell death enhances chemosensitivity to cisplatin in ovarian cancer cell lines and xenografts. Cell Death Dis.

[44] Crighton D, Wilkinson S, O'Prey J, Syed N, Smith P, Harrison PR (2006). DRAM, a p53-induced modulator of autophagy, is critical for apoptosis. Cell..

[45] Fiorini C, Menegazzi M, Padroni C, Dando I, Dalla Pozza E, Gregorelli A (2013). Autophagy induced by p53-reactivating molecules protects pancreatic cancer cells from apoptosis. Apoptosis..

