# Supplementary material for: Re-expression of DIRAS3 and p53 induces apoptosis and impaired autophagy in head and neck squamous cell carcinoma
Source: Mil Med Res. 2020 Oct 11;7:48. doi: 10.1186/s40779-020-00275-3 (PMC7548045; doi:10.1186/s40779-020-00275-3)
Supplement: Supplementary file 1 — Additional file 1: Fig. S1 Expression of GFP in cells treated with Ad-GFP, Ad-DIRAS3, or rAd-p53 alone or with a combination of Ad-DIRAS3 and rAd-p53. [file 40779_2020_275_MOESM1_ESM.pdf]

Additional file

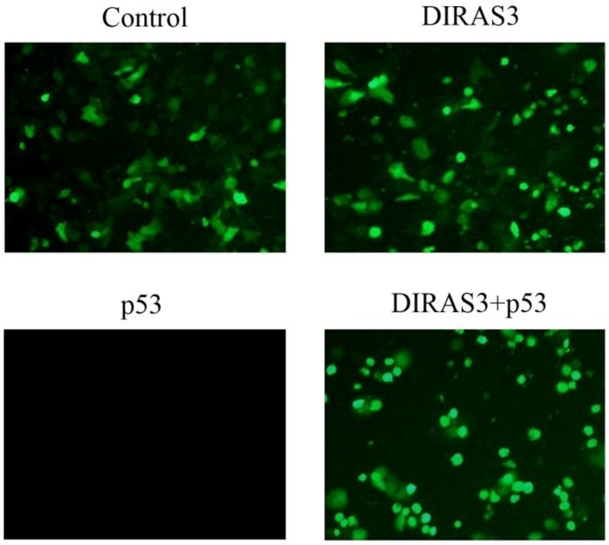

Additional file 1: Figure S1 CAL-27 cells were treated with Ad-GFP, Ad-DIRAS3, rAd-p53, or Ad-DIRAS3 plus rAd-p53 for 24 h. The successful transfer of DIRAS3 or GFP genes after adenovirus infection was confirmed by examining green fluorescence using a fluorescence microscope. No fluorescence was detectable in cells treated with rAd-p53 that do not contain a GFP tag.
